# A case report of mediastinal ectopic parathyroid adenoma presented as parathyroid crisis localized by SPECT/CT

**DOI:** 10.1097/MD.0000000000005157

**Published:** 2016-10-14

**Authors:** Weibin Zhou, Min Chen

**Affiliations:** Department of Endocrinology, the First Affiliated Hospital, College of Medicine, Zhejiang University, Hangzhou, Zhejiang, China.

**Keywords:** ectopic parathyroid adenomas, hypercalcemia, mediastinum, parathyroid crisis, SPECT/CT

## Abstract

**Introduction:**

Parathyroid crisis due to ectopic parathyroid adenomas can pose diagnostic and management challenges, since it is quite rare in clinical practice.

**Clinical Findings/Patient Concerns:**

A 67-year-old Chinese male presented as a parathyroid crisis due to an ectopic mediastinal parathyroid adenoma with his serum calcium and PTH markedly increased in short time. An ultrasonography and computed tomography (CT) scan of the neck did not reveal any parathyroid adenoma. Thoracic CT detected a contrast-enhanced mass in the mediastinum. Although the ectopic location is difficult to appreciate on anterior planar technetium-99m-sestamibi scintigraphy views but has been accurately localized with single photon-emission computed tomography/computed tomography. After fluid resuscitation, loop diuretic, and calcitonin treatment, a thoracoscope surgery was performed. The histopathology of the mediastinal nodule was consistent with a parathyroid adenoma. Hypocalcemia due to hungry bone syndrome occurred after surgery and was resolved quickly with large-dose calcium and calcitriol supplementation. He is asymptomatic and has normal serum calcium and PTH levels on regular follow-up.

**Diagnoses:**

The ultrasonography, CT, sestamibi, and single photon-emission computed tomography/computed tomography provide limited sensitivity in the detecting ectopic parathyroid adenomas alone. The combination of these techniques has incremental value in localizing ectopic parathyroid adenomas over either technique alone.

**Conclusion:**

Any parathyroid crisis without parathyroid adenoma in the neck should alert physicians to search for ectopic locations through combination of imaging techniques.

## 1 Introduction

Parathyroid crisis, also known as parathyroid storm or acute primary hyperparathyroidism, is a rare and serious complication of primary hyperparathyroidism. Fewer than 200 cases have been described in the literature. Prognosis is poor: mortality is 100% in nonoperable cases and 20% in cases even parathyroidectomy is performed. Parathyroid crisis requires aggressive medical therapy and early surgical treatment.^[1]^ Ectopic parathyroid glands continue to be a diagnostic and operative challenge in patients with hyperparathyroidism. Difficulties in locating the ectopic parathyroid adenoma may delay the diagnosis and subsequent surgery. The embryological origin of parathyroid glands is the endoderm of the third and fourth pharyngeal pouches. It is well known that parathyroid glands can be found in aberrant locations, mainly in the thyroid parenchyma or in the mediastinum.^[2]^ Parathyroid crisis due to an ectopic parathyroid adenoma is even rarer, and thus, its diagnosis and treatment can usually be challenging. We present a 67-year-old Chinese male diagnosed of parathyroid crisis due to an ectopic mediastinal parathyroid adenoma with his serum calcium and PTH markedly increased in short time that was localized by the combination of single photon-emission computed tomography/computed tomography (SPECT/CT) and thorax contrast enhanced CT.

## 2 Case report

A 67-year-old Chinese man was brought to the emergency department with 7-day symptoms of fatigue, somnolence, and anorexia. At admission, he had disorientation in time and place, mental confusion, headache, and vomiting. The investigation results showed that serum potassium was 3.1 mmol/L (normal range [N] 3.5–5.5 mmol/L), serum sodium 143 mmol/L (N 136–145 mmol/L), serum calcium 4.57 mmol/L (N 2.08–2.60 mmol/L), ionized calcium 1.82 mmol/L (N 1.3–1.46 mmol/L), phosphorus 0.66 mmol/L (N 0.81–1.62 mmol/L), creatinine 84 μmol/L (N 62–133 μmol/L), and alkaline phosphatase 348 IU/L (N 38–126 IU/L). Several intact serum parathyroid hormone (iPTH) tests were markedly elevated between 303 and 757.7 pg/ml (N 12–65 pg/ml). The other laboratory tests were within the normal range. Electrocardiogram showed sinus rhythm with normal QT interval. He denied any chronic medication or illness, especially renal colic, or peptic ulcers. He didn’t complain of bone pain. The patient has no familial history of primary hyperparathyroidism and/or multiple endocrine neoplasia.

Five weeks before this admission, he was suffered from acute pancreatitis. Therefore he was evaluated at the local hospital. At that time, his serum amylase was 2218U/L (N 32–641U/L), urinary amylase 8835U/L (N 80–300U/L), serum potassium 4.0mmol/L, serum sodium 140 mmol/L, serum calcium 2.49mmol/L, phosphorus 0.47mmol/L, and alkaline phosphatase 70 IU/L. The abdominal CT showed his pancreas was diffuse enlargement with edema. After 12-day treatment, his pancreatitis was relieved and discharged.

**Figure 1:**
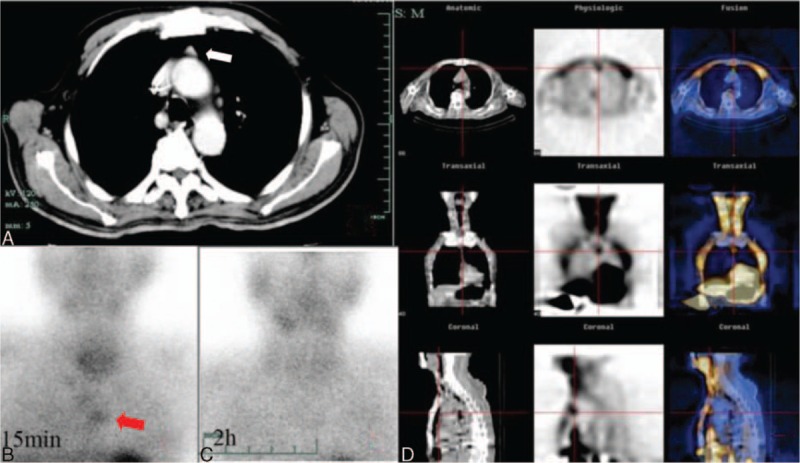
Contrast-enhanced CT of the thorax showed a well-defined soft tissue mass in front of the aortic arch in the upper-anterior mediastinum (A); 99mTc-MIBI showing early tracer uptake by thyroid glands and the gland in superior thorax at 15 minutes (B); rapid 99mTc-MIBI clearance in the superior mediastinum at 2 hours delayed phase (C); the accurate localization of mediastinal ectopic parathyroid adenoma with SPECT/CT (D). CT = computed tomography, MIBI = sestamibi, SPECT = single photon-emission computed tomography.

He was then evaluated in our institution for the persistence of hypercalcemia and parathyroid crisis. His heart, lungs, and abdomen were normal. Liver and spleen were not palpable. There was no lymphadenopathy or palpable mass in the neck. His complete blood cell count, thyroid hormones, cortisone, testosterone, and biomarkers for malignancy were normal. The ultrasonography and CT of the neck did not show any parathyroid adenoma. Thoracic CT detected a well-defined soft tissue mass, measuring 1.36∗0.97∗1.54 cm, in front of the aortic arch in the upper-anterior mediastinum which was contrast enhanced (Fig. 1A). He underwent an anterior planar Technetium-99m-sestamibi (MIBI) scintigraphy scan using a single-tracer, dual-phase technique. Though 99mTc-MIBI showing early tracer uptake by thyroid glands and the gland in superior thorax at 15 minutes (Fig. 1B), rapid 99mTc-MIBI clearance was shown in the thyroid and superior mediastinum at 2 hours delayed phase (Fig. 1C). The accurate localization of mediastinal ectopic parathyroid adenoma was achieved with SPECT/CT (D).

**Figure 2:**
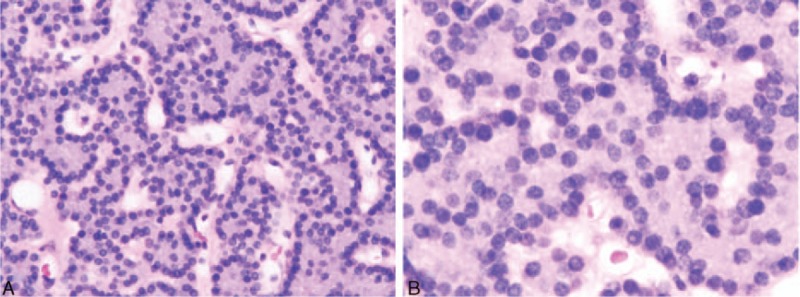
Hematoxylin-eosin stained sections of the adenoma (original magnification ×200 [A] and ×400 [B]). The adenoma was composed mainly of chief cells arranged in a trabecular pattern.

The patient was initially treated conservatively with saline hydration, calcitonin, intravenous use of loop diuretics and hemodialysis. The response was good and serum calcium returned to 3 mmol/L or so. Then a thoracoscopic excision of the mediastinal mass was performed. Histology of the operative specimen confirmed the presence of ectopic parathyroid adenoma. The lesion was composed predominantly of chief cells arranged in a trabecular pattern (Fig. 2). Immunohistochemistry stain reactivity was negative for chromogranin A, synaptophysin, and thyroid transcription factor-1. Serum iPTH and serum calcium returned to normal at 24 hours after surgery. Hypocalcemia and hungry bone syndrome occurred 2 days after surgery and were gradually improved in 2 week with calcium carbonate and calcitriol supplementation. The dose of oral calcium and calcitriol was gradually reduced during follow-up. At present, he is asymptomatic and has normal serum calcium and iPTH levels on regular follow-up.

Written informed consent was obtained from the patient. The study was approved by the Ethics Committee of the First Affiliated Hospital, School of Medicine, Zhejiang University, China.

## 3 Discussion

Parathyroid crisis is an uncommon life-threatening complication of primary hyperparathyroidism and its prognosis is poor; therefore, it requires aggressive medical therapy and early surgical treatment.^[1]^ In the majority of cases, it is a complication of already diagnosed primary hyperparathyroidism. However, in our patient, parathyroid crisis was the first manifestation of primary hyperparathyroidism since the calcium was normal five weeks before this admission. An ectopic parathyroid adenoma that causes primary hyperthyroidism is difficult to detect by various imaging modalities, because ectopic parathyroid adenomas are usually small between 1 and 2 cm.^[3]^ Therefore, ectopic parathyroid adenomas are often detected either in the preoperative work up of patients who present with hypercalcemia or in postparathyroidectomy patient with persistent hypercalcemia.^[4]^ The clinical and laboratory features of patients with primary hyperthyroidism due to ectopic adenomas have been reported to be more severe than those with eutopic adenomas, since the former may manifest with higher calcium levels and more frequent primary hyperthyroidism related bone disease.^[5]^ The localization and treatment of an ectopic parathyroid adenoma that caused parathyroid crisis can usually be challenging.

The base of the tongue to the mediastinum is the most common position for ectopic parathyroid adenomas.^[2]^ The aberrant migration of parathyroid glands in early stages of development may stimulate the formation of ectopic parathyroid glands, while lack of successful identification may cause failure in parathyroid surgery. They attribute to persistent or recurrent hyperparathyroidism consideration of the difficult of their diagnosis at initial stage. The prevalence of ectopic parathyroid adenomas is about 2% to 43% in anatomical series and reaches to 16% and 14% in primary and secondary hyperparathyroidism, respectively.^[2]^ The increasing evidences indicate that higher incidence of ectopic inferior glands is closely related to abnormal migration during embryogenesis.^[6]^ There are 2 superior parathyroid glands developed from the fourth pharyngeal pouch and 2 inferior parathyroid glands derived from the third pouch at the upper two thirds of the posterior surface of the thyroid gland exist in humans.^[7]^ Detached from the pharyngeal wall they follow the descending pathway of the thyroid gland.^[7]^ The inferior parathyroids originate from the dorsal wing of the 3rd pharyngeal pouch and the thymus, which migrates to its final position in the mediastinum, comes from the ventral wing gives. Since the descending route of the inferior parathyroids is similar to that of thymus and this could explain the inferior parathyroids’ unusual location in the mediastinum.^[7,8]^ Ectopic superior parathyroids are often located in the tracheoesophageal groove and retroesophageal region.^[2]^ Parathyroid glands present in a normal anatomic position may enlarge and be displaced to an ectopic location because of lacking capsular fixation. Thus, the pathophysiology for most ectopic superior glands is enlargement and displacement from a normal to an ectopic location.^[6]^

Multiple diagnostic modalities for imaging the parathyroid glands include ultrasonography, CT, magnetic resonance imaging (MRI) and radionuclide scintigraphy.^[3]^ Ultrasonography is commonly used to locate enlarged parathyroid glands due to its convenience and low cost. High-resolution ultrasound can accurately localize adenomas relative to the thyroid gland. However, its ability to detect abnormalities depends on the experience and skill of the operator, and therefore, its sensitivity in localization of enlarged parathyroid glands varies greatly (44%–87%).^[9]^ Just in our case, the ultrasonography didn’t show the lesion.

CT and MRI may further contribute to the identification of ectopic parathyroids and the differential diagnosis from other lesions. CT alone has sensitivity about 65% in detecting parathyroid glands, while the sensitivity of MRI is around 75% to 78%.^[10]^ However, the differential diagnosis of ectopic parathyroids from lymph nodes, thyroid, or thymus tissue depends on the location.

The other most widely used imaging technique, 99mTc-MIBI scintigraphy, has similar sensitivities as ultrasonography. However, when combined with ultrasonography, the overall sensitivity can be increased from 88% to 95%.^[11]^ Dual-phase, dual-tracer 99mTc-MIBI scintigraphy has increased the sensitivity of detection of parathyroid adenomas (up to 90%).^[12]^ High-resolution ultrasound and parathyroid scintigraphy both have their own advantages. The former is good at localizing adenomas relative to the thyroid gland, whereas the latter is usually reserved for detecting adenomas in nodular thyroid disease and at ectopic sites, which incidence is up to 20% of patients.^[13]^ Ectopic parathyroids may be detected with MIBI with almost the same sensitivity as orthotopic adenomas.^[12]^ Focal increased activity separated from the lower pole of thyroid on MIBI pinhole images gives a high probability for ectopic parathyroid adenoma to be located in the thymus.^[14]^ Sestamibi, as a nonspecific tracer that is taken up by mitochondria, may present high uptake in any mitochondria-rich cells. Thus, mitochondrial density is a major factor to determine the retention of 99mTc-MIBI in tissues.^[15]^ There is numerous studies found that the typical retention of 99mTc-MIBI in the parathyroid adenoma was longer than that in the normal thyroid. However, in our case, 99mTc-MIBI showed early tracer uptake by thyroid glands and the gland in superior thorax at 15 minutes and rapid 99mTc-MIBI clearance in the superior mediastinum at 2 hours delayed phase. This was consistent with Benard's reported case of rapid 99mTc-MIBI clearance from a parathyroid adenoma.^[16]^ In that report it is hypothesized that more mitochondrial- rich oxyphil cells in abnormal parathyroid is the critical reason for the prolonged retention of 99mTc-MIBI, because the highest ratio of mitochondria per cell has been found in oxyphil cells.^[17,18]^ A more detailed description is then given of more contributing factors that correlate with the degree of sestamibi uptake, including the size of gland or tumor, the cytological composition, oxyphil cell contents, cell cycle phases and serum calcium levels.^[18]^ More recently, expression of cell membrane proteins, multidrug resistance–associated protein and P-glycoprotein by parathyroid adenomas has been shown to be associated with false negative sestamibi results, although this remains controversial.^[19,20]^ This concept is consistent with our case, because the mediastinal ectopic parathyroid adenoma was only 1 cm in diameter and the histopathology in our case showed hardly any oxyphil cells. Although there is no evidence to show how many oxyphil cells most ectopic parathyroid adenoma have, the absence of these cells and the size of adenoma would explain the lack of 99mTc-MIBI retention on late-phase imaging in our case. Immediate imaging of sestamibi scintigraphy reveals the tracer uptake in superior thorax implied the ectopic parathyroid adenoma combined with CT image and the clinical features of parathyroid crisis. The combination of sestamibi scintigraphy and CT imaging accurately localized the tumor to the anterior mediastinum, thus highlighting the usefulness of combining multiple imaging techniques to locate ectopic active parathyroid gland.

Hybrid imaging with SPECT/CT, which combines scintigraphic datasets (SPECT) and anatomical (CT) has become increasingly valuable over the last few years. The accurate localization of parathyroid adenomas with SPECT/CT to anatomical landmarks should help improve the success of localizing ectopic adenomas just as in our case.

The combination of MIBI with ultrasonography raises the sensitivity to 78% to 96%^[21]^ and with CT or MRI may raise the sensitivity and specificity to 100%.^[22]^ These combinations are cost effective and are indicated as a means for routine preoperative localization of ectopic parathyroid adenomas, especially in cases with faint 99mTc-MIBI scans.^[23]^

The formation of hungry bone syndrome, which is a disease being coined to the rapid, profound, and prolonged hypocalcemia, is extensive remineralization associated with hypophosphatemia after parathyroidectomy.^[24]^ Various risk factors including older age, large parathyroid adenoma, overt bone disease, and vitamin D deficiency are attributed to hungry bone syndrome. Higher bone turnover markers, higher preoperative calcium and PTH levels, and PTH resistance are also involved in the pathology of hungry bone syndrome.^[24]^ In fact, the presence of mild hypocalcemia and hungry bone syndrome reassures the surgeon that the hyperactive adenoma has been successfully removed. In the same time, preoperative treatment with bisphosphonates has been suggested to reduce postoperative hypocalcemia.^[25]^ In our case, hypocalcemia and hungry bone syndrome occurred 2 days after surgery and were gradually improved in 2 week with calcium carbonate and calcitriol supplementation.

## 4 Conclusions

Any hypercalcemia and high level of PTH without parathyroid adenoma in the neck should alert physicians to search for ectopic locations through combination of imaging techniques. The mediastinum should be cautiously noted since it is the very common location for ectopic parathyroid adenoma. Since parathyroid crisis is life threatening and its prognosis is poor, it requires aggressive medical therapy and early surgical treatment.^[1]^ The combination of imaging techniques has incremental value in localizing ectopic parathyroid adenomas over either technique alone.

## Acknowledgments

We would like to thank Jun Yang, Department of Nuclear Medicine, the First Affiliated Hospital, College of Medicine, Zhejiang University for providing the parathyroid SPECT /CT images and Ke Sun, Department of Pathology, the First Affiliated Hospital, College of Medicine, Zhejiang University for providing pathological sections.
